# Cigarette smoke exposure inhibits extracellular MMP-2 (gelatinase A) activity in human lung fibroblasts

**DOI:** 10.1186/1465-9921-8-23

**Published:** 2007-03-12

**Authors:** Giampiero La Rocca, Rita Anzalone, Francesca Magno, Felicia Farina, Francesco Cappello, Giovanni Zummo

**Affiliations:** 1 Sezione di Anatomia Umana, Dipartimento di Medicina Sperimentale, Università degli Studi di Palermo, Via del Vespro 129, 90127 Palermo, Italy

## Abstract

**Background:**

Exposure to cigarette smoke is considered a major risk factor for the development of lung diseases, since its causative role has been assessed in the induction and maintenance of an inflamed state in the airways. Lung fibroblasts can contribute to these processes, due to their ability to produce proinflammatory chemotactic molecules and extracellular matrix remodelling proteinases. Among proteolytic enzymes, gelatinases A and B have been studied for their role in tissue breakdown and mobilisation of matrix-derived signalling molecules. Multiple reports linked gelatinase deregulation and overexpression to the development of inflammatory chronic lung diseases such as COPD.

**Methods:**

In this study we aimed to determine variations in the gelatinolytic pattern of human lung fibroblasts (HFL-1 cell line) exposed to cigarette smoke extract (CSE). Gelatinolytic activity levels were determined by using gelatin zymography for the in-gel detection of the enzymes (proenzyme and activated forms), and the subsequent semi-quantitative densitometric evaluation of lytic bands. Expression of gelatinases was evaluated also by RT-PCR, zymography of the cell lysates and by western blotting.

**Results:**

CSE exposure at the doses used (1–10%) did not exert any significant cytotoxic effects on fibroblasts. Zymographic analysis showed that CSE exposure resulted in a linear decrease of the activity of gelatinase A. Control experiments allowed excluding a direct inhibitory effect of CSE on gelatinases. Zymography of cell lysates confirmed the expression of MMP-2 in all conditions. Semi-quantitative evaluation of mRNA expression allowed assessing a reduced transcription of the enzyme, as well as an increase in the expression of TIMP-2. Statistical analyses showed that the decrease of MMP-2 activity in conditioned media reached the statistical significance (p = 0.0031 for 24 h and p = 0.0012 for 48 h), while correlation analysis showed that this result was independent from CSE cytotoxicity (p = 0.7833 for both exposures).

**Conclusion:**

Present work describes for the first time that, apart well characterized proinflammatory responses, human lung fibroblasts may react to CSE with a significant reduction of extracellular MMP-2 lytic activity. Therefore, fibroblasts may actively participate to the alteration of the proteolysis/antiproteolysis balance, which reflects the defective repair of the extracellular matrix. Such event should provide a further contribution to the maintenance of the inflamed state in the lungs.

## Background

Cigarette smoke is among the major risk factors for the development of chronic lung diseases such as COPD (chronic obstructive pulmonary disease) and emphysema [1]. One of the key features of these diseases is the disruption of the airway wall organisation, followed by an increase in collagen deposition which leads to a progressive loss of lung function [2]. Prolonged exposure to cigarette smoke may lead to an accumulation of macrophages and neutrophils, as observed in pulmonary emphysema, and, as shown for COPD, the inflammatory state is maintained in the disease, even if the cause has been removed (e.g. for smoke cessation after the diagnosis) [1,3]. One of the potential mechanisms for the perpetuation of the inflamed state may involve the control of extracellular matrix (ECM) turnover [4]. ECM is now recognized as an instructive environment for resident and migratory cell types, and not only as a mere molecular scaffold for tissue organisation [5,6]. Since activation of inflammatory cells by cigarette smoke results also in the production of large amount of proteinases, as well as the decrease of inhibitors levels, the global effect is the imbalance of tissue homeostasis [7,8]. Moreover, the generation of proteolytic fragments (matrikins) of ECM molecules by the proteolytic enzymes secreted by different cell types, may contribute to prolong the effects of inflammation even after the cessation of the causative stimulus. This process may take place by the recruiting activity of ECM fragments towards neutrophils and monocytes, but also by the activation of growth/survival factors triggering inflammation [9-11]. Matrix degrading proteinases belong to different classes, grouped on the basis of their catalytic features. In particular, matrix metalloproteinases (MMPs) constitute a broad family of more than 20 members, which share a significant structural homology and domain organisation and feature a zinc ion binding site into their catalytic domain [12,13]. Different subgroups of MMPs have been characterised, on the basis of their substrate specificity (e.g. collagenases, elastases and gelatinases), even if different enzymes may also share similar substrates. This overlap of target molecules, both ECM structural proteins and regulatory ones, reflects the complex organisation of matrix microenvironmental regulation. Gelatinases, also named Type IV collagenases, are two enzymes (MMP-2 or gelatinase A and MMP-9 or gelatinase B) which play a key role in a number of physiological processes. In particular, in lung ECM biology, these molecules are involved in developmental processes, as well as in tissue repair and fibrosis [14]. Moreover, their role has been highlighted in several pathological conditions, as asthma, COPD and lung cancer. Further emerging evidences indicate that the cross-talk between different cell types and extracellular matrix molecules should be considered a determining factor influencing the outcome of inflammatory events. Furthermore, damaging external stimuli (as airborne pollutants and smoke components inhaled with respiration) can provide a variety of signals which can induce an inflammatory response. Indeed, CSE-treated lung fibroblasts are able to secrete chemotactic molecules for both neutrophils and macrophages [15,16]. Moreover, fibroblasts stimulated by CSE may produce prostaglandin, thereby contributing to the creation of a proinflammatory microenvironment [17]. Furthermore, as recently stated, gelatinases produced by structural cells (as demonstrated for mouse lung fibroblasts) may play a role in the pathogenesis of COPD, and this process seems to be regulated by CSE exposure [18]. In the lung matrix, fibroblasts regulate the composition of the ECM scaffold, by deposing new collagen and elastin molecules, both in the physiological turnover of matrix and in the reparative processes following injuries. Instead, as occurs in smoke-driven persistent inflammation, the general mechanisms of tissue repair fail, and matrix alterations may accumulate, leading to a diseased state. In this process, the correct balance between proteinases and inhibitors is critical for tissue repair and remodelling [19]. The use of cigarette smoke as source of damage for lung cells allows mimicking the effects that may take place in vivo as a consequence of smoking and, also in animal models, smoke exposure is currently considered the best model for COPD development [20]. Moreover, CSE has been widely used to investigate smoke effects in both oxidative and inflammatory processes in pulmonary cells [21]. Therefore, in the present study we aimed to determine the variations in the gelatinolytic pattern of cultured human lung fibroblasts exposed to increasing concentrations of CSE. The observed modulator effect of CSE on fibroblast-secreted gelatinases may in part explain the effects due to cigarette smoke exposure in vivo, and confirm the recent hypotheses on the central role of fibroblasts in the development of chronic lung diseases.

## Methods

### Cell cultures

Human foetal lung fibroblasts (HFL-1, lung, diploid, human) were obtained from ATCC. This cell line was initiated from the lung tissue of a 16–18 week old human foetus. The cells retain morphological features and expression pattern of lung fibroblasts (including collagen and fibronectin production) [22]. Cells were cultured in Dulbecco's modified Eagle's medium (DMEM) containing 10% foetal calf serum (FCS), 100 U/ml penicillin, 100 μg/ml streptomycin, 0.25 μg/ml amphotericin B and 2 mM L-glutamine (all from Invitrogen, Milan). Cultures were maintained at 37°C in a humidified atmosphere of 5% CO2. Cells from passages 17–22 were used for experiments.

### Preparation of cigarette smoke extract

Cigarette smoke extract (CSE) was prepared by a modification of the method of Carnevali et al [23]. In brief, two cigarettes without filters were combusted with a modified vacuum-driven apparatus. The smoke was bubbled through 50 ml of serum-free DMEM, and the suspension was then adjusted to pH 7.4 and filtered through a 0.22-μm pore filter. This medium was defined 100% CSE and was applied to fibroblast cultures at different percentages (1, 2, 5 and 10%) within 20 min of preparation. Exposure of cells to CSE was carried out with incubation times of 24 and 48 hours.

### Cell viability assay

Cell viability after CSE exposure was evaluated by MTT [3-(4,5-dimethylthiazol-2-yl)-2,5-diphenyltetrazolium bromide] assay. MTT (Sigma-Aldrich) is a yellow water-soluble tetrazolium dye which is reduced in live cells to a water-insoluble dark blue formazan precipitate by a mitochondrial dehydrogenase enzyme. Briefly, after CSE exposure for 24 or 48 h in 24-well plates, culture medium has been replaced by MTT (diluted in phenol-red free DMEM). After an incubation period of 3 h, the solubilisation of the precipitate from cells was accomplished by using acidic absolute isopropanol (HCl 0.04 M). The amount of formazan was therefore determined spectrophotometrically by reading A_570_, with background subtraction at 650 nm. Triton X-100 (1% v/v) was used as a positive control for cytotoxicity. Triplicate assays were performed using cells at different culture passages.

### Gelatin zymography

Gelatin zymography was carried out for both conditioned media and cell lysates as described previously [24]. In brief, gels (SDS-PAGE, 7.5%) were copolymerised with gelatin (0.1% w/v) (Sigma-Aldrich). Following constant voltage electrophoresis, gels were washed in renaturation buffer (2.5% Triton X-100 in 50 mM Tris-HCl pH 7.5) for 1 h in an orbital shaker. Then the zymograms were incubated for 18 h at 37°C in incubation buffer (0.15 M NaCl, 10 mM CaCl_2_, 0.02% NaN_3 _in 50 mM Tris-HCl pH 7.5). Gels were then stained with Coomassie blue and destained with 7% methanol and 5% acetic acid. Areas of enzymatic activity appeared as clear bands over the dark background.

### Assays for the in vitro inhibition of CSE on gelatinase activity

In order to assess the effects of direct chemical inhibition of cigarette smoke extract on gelatinolytic activities revealed by subsequent zymography, in vitro incubation of different samples containing gelatinases was performed. We used different samples containing gelatinase activities of both MMP-2 and MMP-9, and in particular native human serum, and MMP-Control-1 (Sigma-Aldrich). Incubation was carried out overnight at 37°C and gelatin-degrading activity was evaluated by subsequent gelatin zymography and, after Coomassie blue staining, densitometric quantification of lytic bands was performed as described below.

### Identification of gelatinases by means of specific chemical inhibition

To assess the identity of the lytic bands present in conditioned media and cell lysates zymographies, parallel experiments of zymographic inhibitions were performed. Addition of EDTA (20 mM) or 1,10-Phenantroline (Sigma-Aldrich) (5 mM) to the incubation buffer was used in order to inhibit lytic activities due to metalloproteinases (as gelatinase A and B), while PMSF (inhibitor of serine proteinases) (Sigma-Aldrich) was used to inhibit activities due to serine proteinases.

### Semi-Quantitative evaluation of lytic activity

Following zymography, the degree of gelatin digestion was quantified using a scanner equipped with a transparency adapter interfaced to an IBM PC. Gels were scanned using 1D ScanEX software, version 3.1 for Windows (Scanalytics), in a grey scale mode. The image was digitally inverted, so that the integration of bands was reported as positive values. The pixel density was determined after background subtraction and used to calculate the integrated density of a selected band. Values of integrated density were reported in volume units of pixel intensity per mm^2^. The integrated density of each band is reported as the mean of three different measurements of the same band for each sample run in triplicate. Therefore, for both conditioned media and cell lysates, we performed triplicate zymographic runs using samples from cells at different passages. Moreover, for each experiment, the intensity of each lytic band was measured three times in order to exclude software-driven measurement errors. Data plotted in the final graphs represent the mean and standard deviation of the three mean values obtained for each experimental set.

### Statistical analyses

Data from different experiments were plotted using MS Excel software. Statistical analyses were performed using GraphPad Prism 4 software (GraphPad Software, San Diego, USA). The statistical methods used were nonparametric analyses. In particular, significance of differences of activity levels of MMP-2 between controls and CSE treatments was assessed by Kruskal-Wallis test, and correlation analyses between different variables were performed using the Spearman method. Values were considered significant for p < 0.05.

### Total RNA extraction

Total RNA extraction was accomplished using the QuickPrep Total RNA Extraction Kit (Amersham Biosciences, Milan) following the manufacturer's instructions. RNA yield was evaluated spectrophotometrically (A260/A280) and RNA aliquots were stored at -80°C until use. Total RNA fractions were used for subsequent experiments only if the A260/280 ratio was in excess than 1.6.

### Conventional qualitative RT-PCR

RT-PCR was performed using the Ready To Go RT-PCR beads (Amersham Biosciences). The reaction was carried out using the two step protocol provided with the kit, with a MyCycler thermal Cycler with gradient module (Bio-Rad, Milan). RT-PCR was carried out mixing 1 μg of total RNA, 0.5 μg of pd(T)_12–18_, 1 μg of pd(N)_6_, with RNAse free water. The reaction comprised a reverse transcription step of 30 minutes (42°C), followed by inactivation of the enzyme at 95°C (5 min). Then 100 pM of specific primers were added and the reactions were cycled for 95°C, 2 min, then 35 cycles of 95°C, 60 s, 60°C, 60 s, 72°C, 60 s, with a final extension at 72°C, 10 min.

Primers used in this study were as follows:

MMP-2 Forward: 5'-TGATGGTGTCTGCTGGAAAG-3';

MMP-2 Reverse: 5'-GACACGTGAAAAGTGCCTTG-3'; product size 280 bp

MMP-9 Forward: 5'-CATTTCGACGATGACGAGTTG-3';

MMP-9 Reverse: 5'-AAGCCCCACTTCTTGTCGCT-3'; product size 554 bp

GAPDH Forward: 5'-AAGGTGAAGGTCGGAGTCAA-3';

GAPDH Reverse: 5'-AAGTGGTCGTTGAGGGCAAT-3'; product size 914 bp

YWHAZ Forward: 5'-TTGGCAGCTAATGGGCTCTT-3';

YWHAZ Reverse: 5'-TCTGTGGGATGCAAGCAAAG-3'; product size 515 bp

Beta Actin Forward: 5'-AAACTGGAACGGTGAAGGTG-3';

Beta Actin Reverse: 5'-TCAAGTTGGGGGACAAAAAG-3'; product size 350 bp

TIMP-1 Forward: 5'-GCTGACATCCGGTTCGTCTA-3';

TIMP-1 Reverse: 5'-CAGGCTTCAGTTCCACTCCG-3'; product size 418 bp

TIMP-2 Forward: 5'-CTCTCCATTTGGCATCGTTT-3';

TIMP-2 Reverse: 5'-ACTCTTGTGTGTTCCCAGCA-3'; product size 278 bp

TIMP-3 Forward: 5'-CAAGGGGCTGAACTATCGGT-3';

TIMP-3 Reverse: 5'-CATTGATGATGCTTTTATCGG-3'; product size 495 bp

MMP-14 Forward: 5'-GTGCCCTATGCCTACATCCG-3';

MMP-14 Reverse: 5'-GCAGCATCAATCTTGTCGGTA-3'; product size 770 bp

TBSP-1 Forward: 5'-AATTGCAAAGAAAGCCATGA-3';

TBSP-1 Reverse: 5'-TTTCAATGCTATTTCCTTATTGG-3'; product size 306 bp

TBSP-2 Forward: 5'-GATGGATAGGGGGCAAATCT-3';

TBSP-2 Reverse: 5'-CATCATCGTCACTCCCACAC-3'; product size 316 bp

Beta Actin was chosen as housekeeping gene, over GAPDH and YWHAZ, for the better linearity of expression in all the experimental conditions (data not shown).

The identity of PCR products has been confirmed by incubation with the appropriate restriction enzyme and subsequent visualisation of the cleavage products on 2% agarose gel.

### Semi-quantitative evaluation of mRNA expression

In order to determine quantitative differences in the expression of mRNAs of MMP-2 as well as TIMP-2 and the two thrombospondin molecules, we performed densitometry analysis on multiple RT-PCR experiments, using cells at different culture passages, with subsequent normalization of the band intensities for a housekeeping gene (beta actin) [16]. Briefly, after total RNA extraction and DNAse treatment, reverse transcription was accomplished by using the JumpStart Red HT RT-PCR kit (Sigma-Aldrich), following the manufacturer's instructions for a two step protocol. The cycles of PCR reaction were as follows: 94°C, 2 min, then cycles of 94°C, 15 s, 59°C, 30 s, 72°C, 60 s, with a final extension of the product at 72°C, 10 min. The products of interest were visualized on 2% agarose gels, stained with ethidium bromide, and densitometry was performed in order to calculate the normalized band intensity for each PCR product at the different experimental conditions. Kruskal-Wallis test was used to assess the statistical significance of differences with respect to CSE exposure.

### Semi-quantitative multiplex PCR (SM-PCR)

In order to confirm the quantitative differences in the expression of of MMP-2 mRNA following CSE exposure, we used the protocol described by Spencer and Christensen [25] and further improved by other authors [26,27], with minor modifications. Briefly, after reverse transcription we used a range of cycles starting from the minimum number necessary to visualize the product on 2% agarose gel. The cycles were as follows: 94°C, 2 min, then cycles of 94°C, 15 s, 59°C, 30 s, 72°C, 60 s, with a final extension of the product at 72°C, 10 min. The cDNA of MMP-2 was co-amplified with beta actin cDNA over a range of cycles in the exponential phase of the PCR reaction (maintaining the intensity of the band of beta actin constant by adding the related primers at the appropriate number of cycles before the end of the reaction). After 2% agarose electrophoresis, the bands corresponding to the MMP-2 amplification product were quantified using the 1D Scan EX software and, after normalisation for those of actin, the intensities were plotted as function of cycle number. GraphPad Prism software package was used to calculate the exponential regression equations fitted to the curves. Calculation of the R^2 ^values was performed to ensure that the range of cycles used was in the exponential phase of the amplification reaction.

### Affinity chromatography purification of gelatinases

Affinity chromatography has been performed as described previously [24]. Conditioned media from serum-free cultured cells were dialysed against equilibration buffer (50 mM Tris-HCl, 1 M NaCl pH 7.5). The resin (Gelatin-Sepharose 4B, Amersham Biosciences) was rinsed thoroughly in the same buffer. Then, the sample and resin were placed in a tube (batch procedure) and mixed for 3 h at 4°C with mild inversion. Subsequently, resin was briefly centrifuged in order to recover the supernatant (unbound fraction) and successive washes with equilibration buffer were performed to remove all unbound proteins. All the steps were monitored by measuring A280 using an Ultrospec 1000 photometer (Amersham Biosciences). Elution of gelatinases was accomplished by using a 10% DMSO solution; fractions were then dialysed against MilliQ water for lyophilisation and western blotting analysis.

### Western blotting

After electrophoresis (SDS-PAGE 8%), proteins were transferred on PVDF (Poly Vinylidene Di Fluoride) membrane (Amersham) using a semi-dry system (Bio-Rad). After blotting, blocking was performed with 5% non-fat milk in T-PBS (0.05% Tween-20 PBS) for 1 hour at room temperature. Incubation with the primary antibody was performed with anti MMP-2 (Chemicon cat. MAB13406, clone VB3, 1 μg/ml), anti MMP-9 (Calbiochem clone GE-213, 1:400), anti TIMP-2 (Chemicon cat. MAB 13432, clone T2-101, 1 μg/ml) diluted in 1% BSA T-PBS, and was conducted overnight at 4°C. After appropriate washings (six times for five minutes each), secondary antibody (anti-mouse HRP-linked, Amersham, 1:10000 in 1% BSA T-PBS) was incubated with the membrane. After a second washing step, the membrane was incubated with the chemiluminescence substrate (ECL Amersham) and exposed to an autoradiographic film (Kodak BioMax).

## Results

### CSE exposure of HFL-1 cells and cell viability

**Figure 1:**
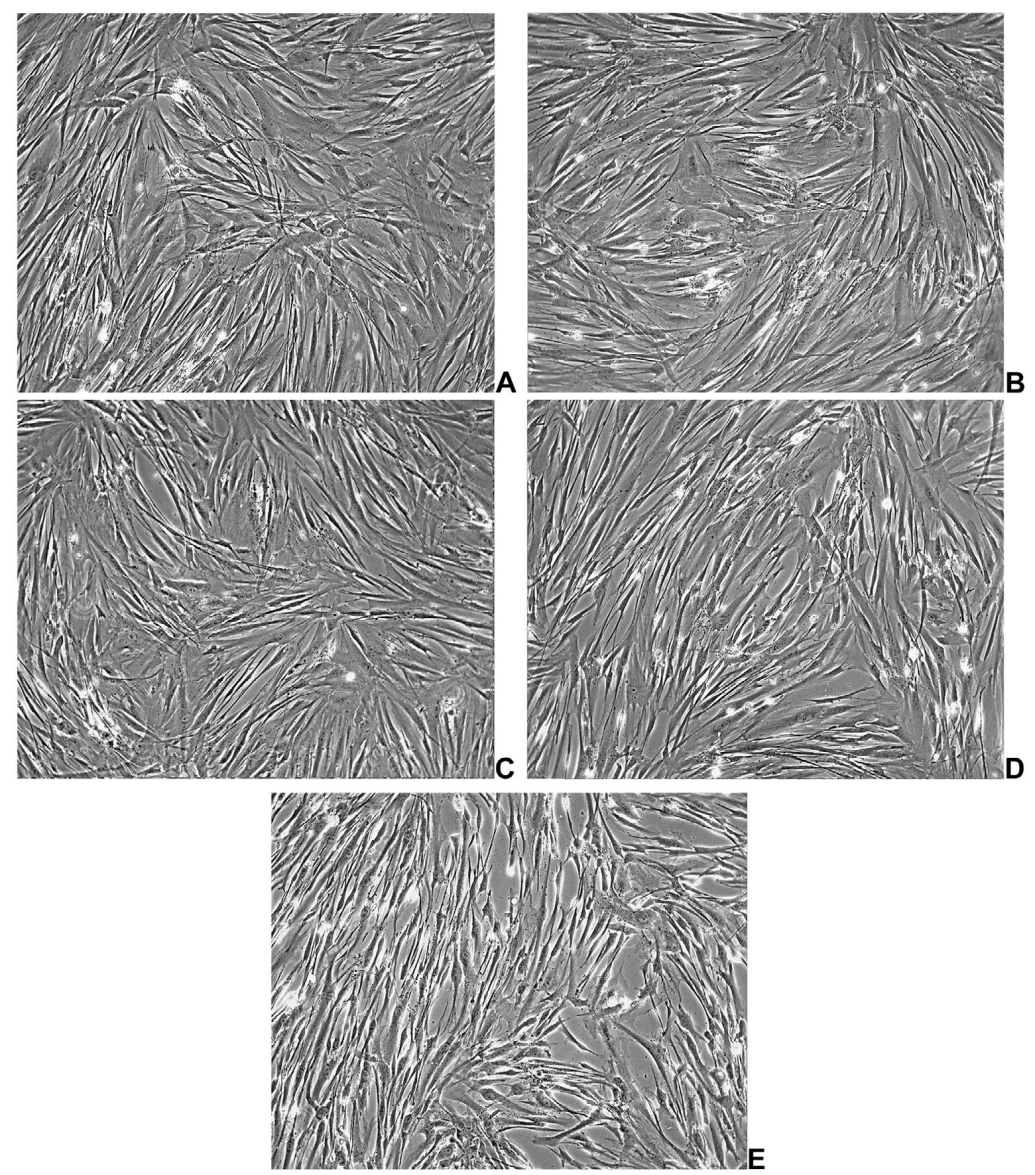
**Panel of representative phase contrast micrographs of HFL-1 cells after 24 hours of CSE incubation**. A) Untreated Control; B) 1% CSE; C) 2% CSE; D) 5% CSE; E: 10% CSE; F: 20% CSE. Magnifications 20×.

**Figure 2:**
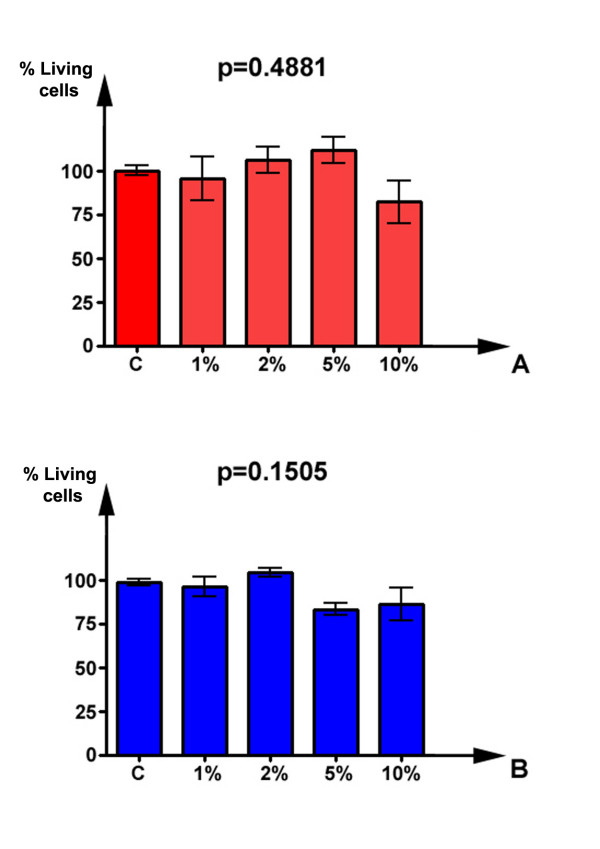
**Cytotoxicity assay of HFL-1 cells after exposure to CSE for 24 and 48 hrs**. Data are represented as mean (with the indication of SEM) of three replicate experiments. Statistical significance of differences has been evaluated by Kruskal-Wallis test.

Exposure of HFL-1 fibroblasts to aqueous extract of cigarette smoke was accomplished after a starvation period of 24 h, then exposing cells to increasing (1–10%) concentrations of CSE for 24 h and 48 h. As visible in figure 1, CSE determined little effects on morphologic features of lung fibroblasts at low concentrations, without relevant phenotypical changes. Parallel viability assays were performed by using MTT test, and the results are schematised in figure 2. As shown, and in good agreement to morphological evaluation, the doses of CSE used in this study did not result in a significant increase of cell death, at both 24 h (p = 0.4881) and 48 h (p = 0.1505) treatment (Kruskal-Wallis test). The concentrations of smoke extract used for this study were in the range of those used in previously published studies. In fact, Ten Hacken and colleagues have recently reviewed [21] the in vitro models of acute exposure to cigarette smoke: their analysis indicated that the numbers of cigarettes used, as well as the exposure times, varied considerably (e.g. the number of cigarettes used varied between 8 × 10^-5 ^and 4 cigarettes/ml). In the present work, we kept the experimental exposure of cells in the lower range of concentrations, with a maximum of 4 × 10^-3 ^cigarettes/ml (achieved with the 10% CSE dose).

### In vitro inhibition of gelatinase activity by CSE

**Figure 3:**
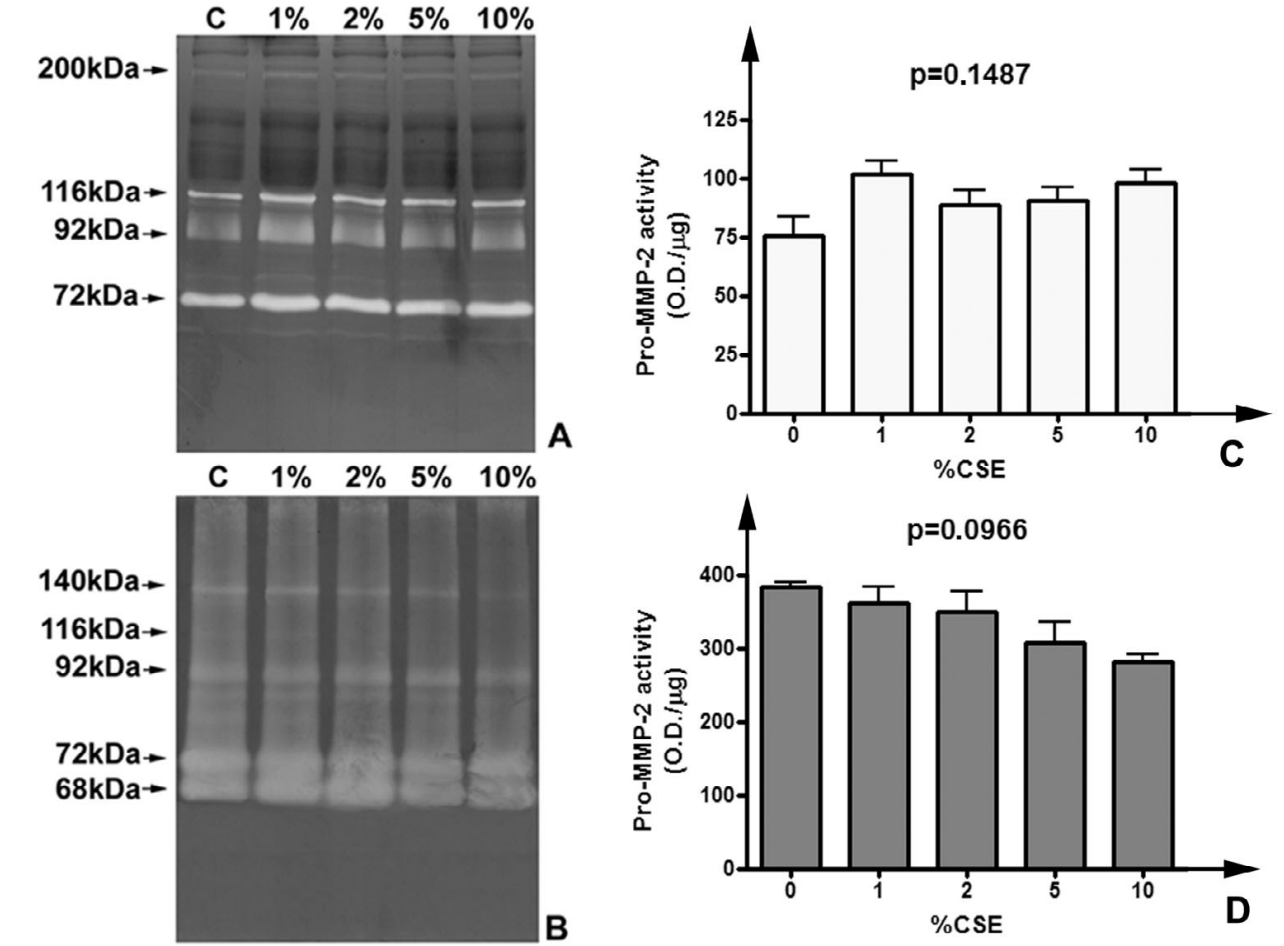
**CSE exposure does not inhibit gelatinase A activity in vitro**. Representative zymograms of gelatinase standards incubated with CSE for 24 h. A) Human native serum samples (28 μg total proteins/lane) incubated overnight with growing concentrations of CSE. B) MMP-Control-1 standard of gelatinases (2 μl/lane) subjected to the same treatment. C) Densitometric analysis of pro-MMP-2 activity levels in human serum exposed to CSE. Data are normalized for protein loading and are represented as mean (with indication of the standard deviation) of three replicate experiments. Significance of differences has been calculated with the Kruskal-Wallis test. D) Densitometric analysis of pro-MMP-2 levels in MMP-control-1 after CSE exposure.

Zymographic methods represent a reliable technique to evaluate lytic activity of ECM-degrading enzymes [28]. Since to our knowledge no studies have been previously performed to assess the direct effects of cigarette smoke extracts on gelatinolytic activity of MMP-2 and MMP-9, (e.g. any form of direct chemical inhibition due to one or more constituents of smoke), we initially performed in vitro assays to determine possible effect of CSE on gelatinase activities from different sources. As shown in figure 3, different samples containing gelatinases (human serum and a commercially available MMP standard, obtained from conditioned medium of stimulated fibroblasts) were incubated overnight with CSE at 37°C, using the same concentration range of cell stimulation experiments (1–10%). After incubation, samples were subjected to gelatin zymography (which allows detecting gelatinolytic activity as negatively-stained clear bands) and subsequent semi-quantitative evaluation of lytic activity. As visible in figure 3(A,C), experimental concentrations of CSE did not inhibit significantly (p = 0.1487) the activity of gelatinase A, for both the proform (72 kDa) and active enzyme (68 kDa), whereas such an effect may be hypothesized for some of the higher molecular weight complexes in MMP-Control-1 (for example the 140 kDa band, an oligomeric complex of MMP-2) (Figure 3B). However, the decrease of pro-MMP-2 activity levels in MMP-control-1 failed to reach the statistical significance (p = 0.0966) (Figure 3D). Therefore, the working concentrations of CSE used in our experiments appeared not causing any decrease in proMMP-2 activity levels. Interestingly, some higher molecular weight complexes appeared to be inhibited in a proportional fashion by smoke extract, even if it is not clear if it should be due to the breaking of the interaction in the etherodimer. Moreover, since an apparent linear trend of decrease of MMP-2 should be suggested by densitometry (Figure 3D), a non-parametric trend test (Jonckheere-Terpstra). The results of the test indicated that the trend did not reach the statistical significance (p > 0.05).

### Gelatin zymography of conditioned media and densitometric evaluation of pro-MMP-2 activity levels

**Figure 4:**
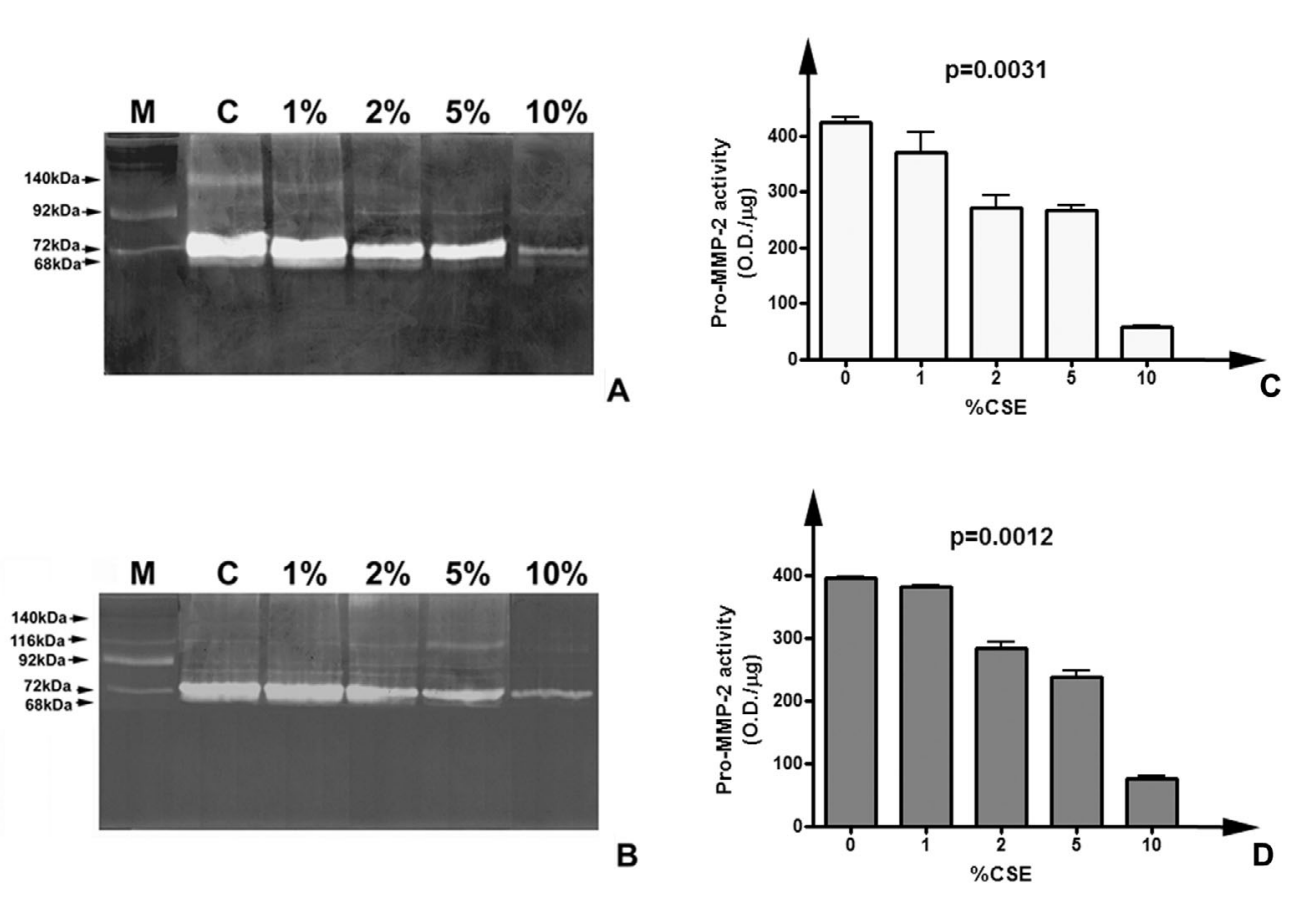
**Gelatinolytic pattern of HFL-1 fibroblasts conditioned media after CSE exposure**. Panel of representative zymograms of conditioned media (10 μg/lane) of HFL-1 cells after 24 (A) and 48 (B) h of CSE exposure. M) Standard of human serum showing the two progelatinase bands. C) Densitometric analysis of pro-MMP-2 activity levels in conditioned media of HFL-1 cells after 24 h CSE exposure. D) Densitometric analysis pro-MMP-2 activity levels in conditioned media of HFL-1 cells after 48 h CSE exposure.

Gelatin zymography experiments allowed characterising gelatinolytic activities in conditioned media and cell lysates of cultured fibroblasts. Figure 4(A,B) shows the representative zymographic panels of conditioned media from CSE-treated and untreated HFL-1 lung fibroblasts after 24 h and 48 h incubation. Samples were dialyzed against milliQ water, lyophilized and resuspended in MilliQ water previous to perform electrophoresis. Samples were loaded in equal amounts (10 μg total proteins/lane) in order to perform subsequent semi-quantitative evaluation of lytic activities. Moreover, triplicate experiments were performed using cell cultures at different passages to minimize the effects due to sample loading or gel staining errors. As shown (figure 4A–B), untreated cells expressed prominently pro-MMP-2 (72 kDa) both after 24 and 48 h of culturing in serum free medium. Activated form of MMP-2 (68 kDa) was present as a faint band, suggesting that no activation process was taking place. Moreover, higher molecular weight bands indicated the presence of putative oligomeric complexes probably due to binding of gelatinase to inhibitory molecules (as TIMPs). As shown, smoke exposure caused a variation in the gelatinolytic pattern, both qualitative and quantitative. The degree of substrate degradation was reduced in parallel with increasing concentrations of CSE, both for 24 h and 48 h exposures. The activity attributable to pro-MMP-2 decreased with the increase of CSE concentration in the culture medium, and the higher molecular weight complex at 140 kDa quickly disappeared, so that at 10% CSE it was no more detectable. Histograms in figure 4(C,D) represent the results of semi-quantitative densitometry of lytic activity of progelatinase A in conditioned media of HFL-1 cells. Densitometric analysis was performed on triplicate zymograms (for both 24 and 48 h treatments) from conditioned media at different culture passages, and following staining each band was evaluated three times, in order to minimize the occurrence of experimental errors. Data are represented as means of the replicated experiments, with the indication of the standard deviation. As indicated, values of activity levels were also normalised for protein loading and are represented as O.D./μg of loaded proteins. The trend of decrease of lytic activity level of pro-MMP-2 is detectable both after 24 and 48 hours of exposure to CSE, with respect to untreated cells. Higher concentrations of CSE corresponded to lower values of MMP-2 activity. Statistical analysis (Kruskal-Wallis test) showed that the effect of CSE on decrease of lytic activity of gelatinase A was statistically significant (p = 0.0031 for 24 h and p = 0.0012 for 48 h). In order to exclude that the effects detected on MMP-2 activity levels should derive merely from cell death due to CSE exposure, a non-parametric correlation analysis was performed between MMP-2 levels and cellular viability. For both conditions, there was no correlation (p = 0.7833 for both exposure times) therefore the effects of CSE on extracellular lytic activity of progelatinase A were independent by cytotoxicity of this extract and should be attributable to a direct regulatory effect of smoke on lung fibroblasts.

### Gelatin zymography of cell lysates

**Figure 5:**
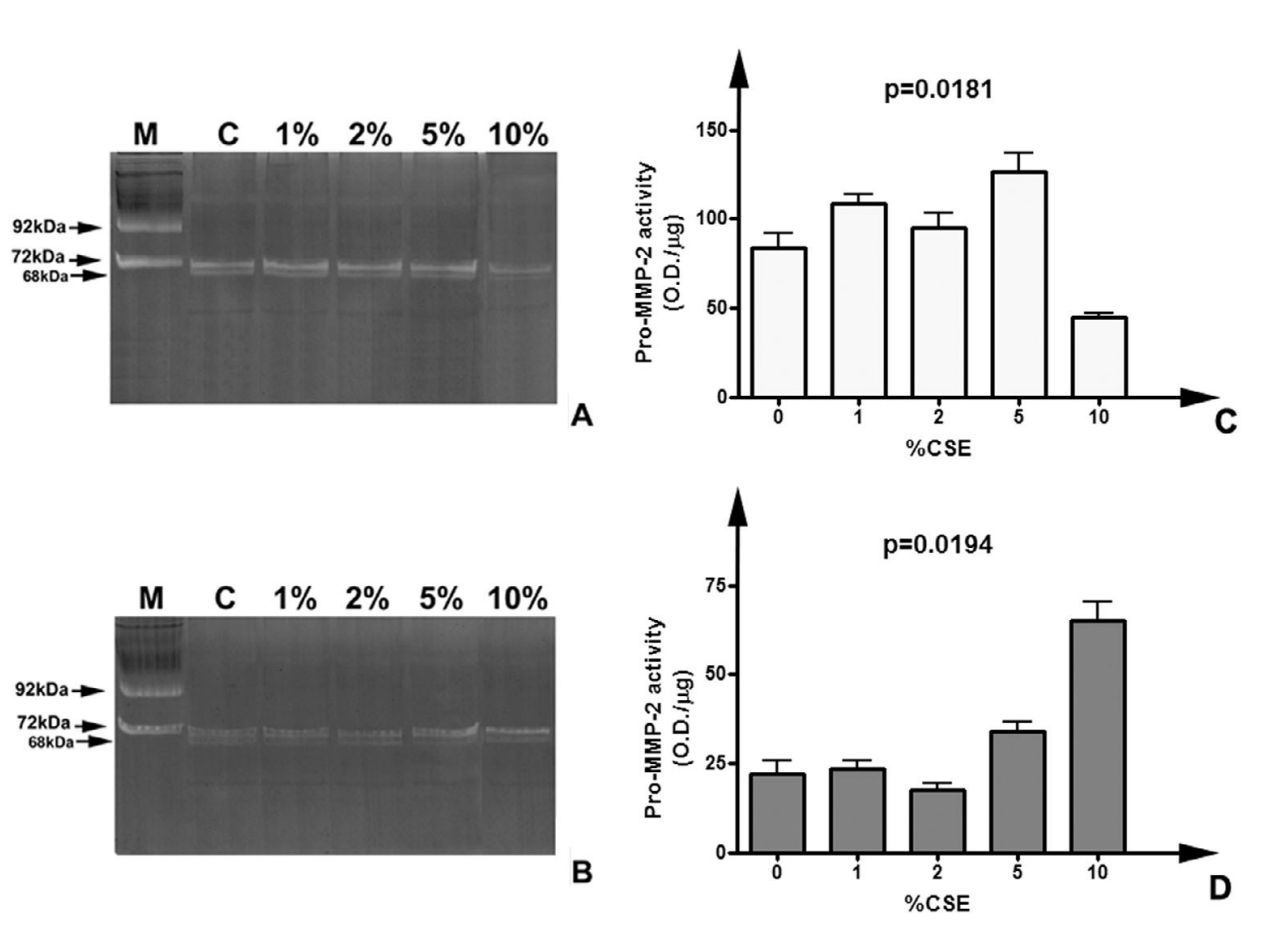
**Gelatinolytic pattern of HFL-1 cell lysates following CSE treatment**. Panel of representative zymograms of HFL-1 cell lysates (20 μg/lane) after 24 (A) and 48 (B) h of treatment. M) Standard of human serum showing the two progelatinase bands. C) Densitometric analysis of pro-MMP-2 activity levels in cell lysates after 24 h CSE treatment. D) Densitometric analysis of pro-MMP-2 activity levels in cell lysates after 48 h CSE treatment.

In order to assess the effects of smoke on activity levels of MMP-2 in the cell layer, parallel experiments were performed in triplicate subjecting cell lysates to gelatin zymography. As shown in figure 5, which represents random gels selected from triplicate experiments at both 24 and 48 h incubation, pro-MMP-2 (together with its active form) was detectable in all experimental conditions. Moreover, densitometry of the lytic bands allowed determining the significance of differences between the experimental conditions. In particular, as shown in figure 5C, after 24 h treatment pro-MMP-2 activity decreased mainly at 10% CSE, while at lower doses its levels were higher than control (p = 0.0181). For 48 h exposure, the levels of MMP-2 activity increased significantly at 5% and 10% CSE (p = 0.0194) (figure 5D). Therefore, the decrease of progelatinase A extracellular activity was only in part matched by the intracellular levels of the enzyme. Moreover, a reduction in the baseline levels of activity of MMP-2 in 48 h cell lysates, with respect to 24 h, should be noted. We could hypothesize that this effect, independent by CSE treatment, should be due to the long (72 h comprising starvation) period of unphysiologic serum deprivation. Therefore we decided to perform most of the subsequent experiments after a 24 h CSE exposure.

### Identification of MMP-2 forms in conditioned media

**Figure 6:**
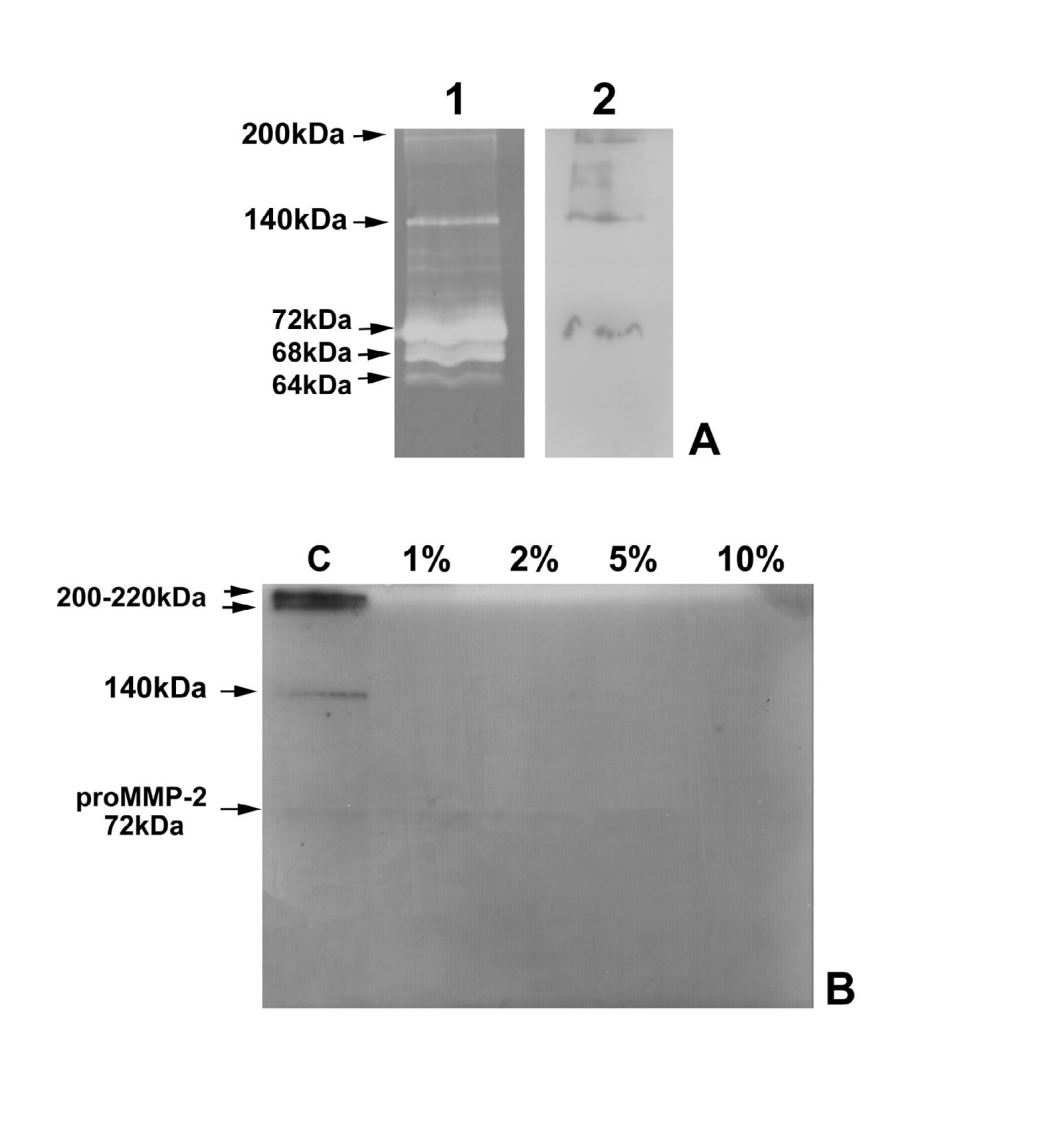
**Characterisation of MMP-2 forms and complexes in conditioned media**. A: identification of zymographic activities in hfl-1 conditioned medium. 1) Zymographic analysis of chromatography-purified conditioned medium of untreated HFL-1 cells; 2) Western blotting of chromatographically-purified HFL-1 conditioned medium with anti-MMP-2 antibody. B: Western blotting with anti-MMP-2 antibody on conditioned media of HFL-1 cells after 24 h treatment. Loading 30 μg/lane.

To assess the identity of the lytic bands detected after zymography of conditioned media and cell lysates, parallel experiments of zymographic inhibition were performed. Addition of EDTA (20 mM) or 1,10-Phenantroline (5 mM) to the incubation buffer totally inhibited the lytic activity in both conditioned media and lysates (not shown), confirming the identity of the enzymes as metalloproteinases, while PMSF (inhibitor of serine proteinases) did not show significant inhibitory activity on all the lytic bands (data not shown). Moreover, after chromatographic purification on gelatin-sepharose, as shown in figure 6A, the gelatinase-enriched fraction of conditioned medium of untreated fibroblasts was subjected to immunoblotting with anti-MMP-2 antibody. As shown, three of the lytic bands present in the parallel zymography (MW 72, 140 and 200 kDa) were recognised by the antibody, showing that two of the higher molecular weight activities are represented by complexes formed by MMP-2 and other molecular partners, probably with inhibitory activity towards gelatinase A. Moreover, as shown in figure 6B, western blotting analysis performed on unfractionated conditioned media after 24 h treatment confirmed the results of zymography, since only in conditioned medium from untreated cells were detectable the higher molecular weight complexes of MMP-2 (140, 200 and 220 kDa), while the proenzyme seemed to decrease with the increase of CSE concentration. Parallel experiments using an anti-MMP-9 antibody gave negative results (not shown), therefore this enzyme was not detectable in its proenzymatic form or in higher molecular weight complexes under present experimental conditions. Moreover, parallel western blotting analyses using an anti-TIMP-2 antibody gave negative results; therefore the nature of the molecular partners of MMP-2 in higher molecular weight complexes remains to be determined.

### Expression analysis of mRNAs for gelatinases and TIMPS: qualitative RT-PCR

**Figure 7:**
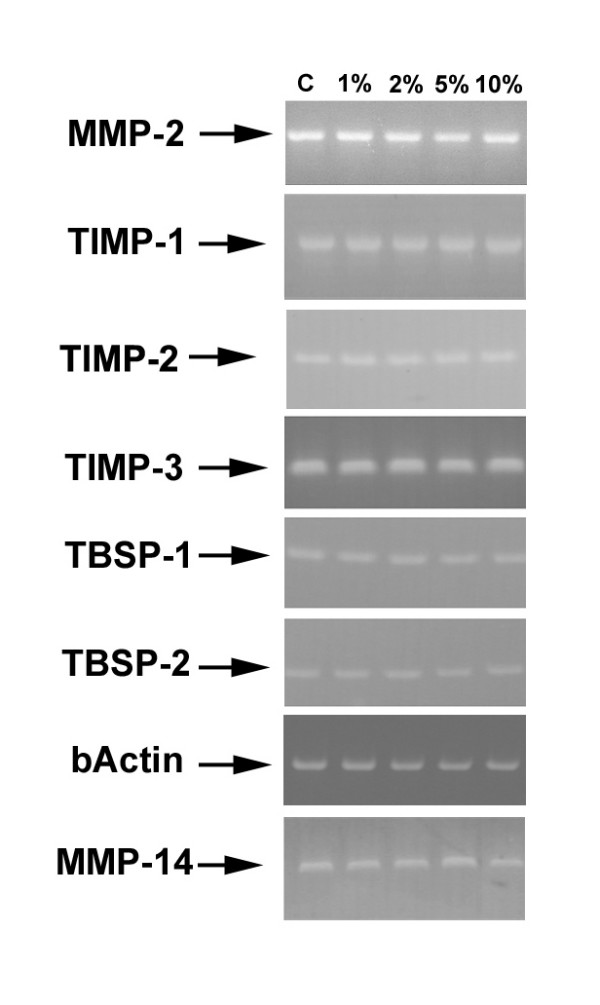
**Qualitative RT-PCR analysis for the expression of gelatinases and TIMPs in treated HFL-1 fibroblasts**. Qualitative RT-PCR was performed as stated in materials and methods. Loading 20 μl/lane. Cycling of 35 cycles for each product.

In order to achieve information regarding the expression of MMPs and their inhibitors in HFL-1 cells following smoke challenge, total RNA was extracted from cells and RT-PCR was performed using gene-specific primers. As schematised in figure 7, MMP-2 mRNA was detected in all the experimental conditions after 24 h treatment. Moreover, MMP-9 messenger was not detectable, thus confirming the lack of the molecule suggested by zymography and western blotting analyses. Regarding inhibitors of MMPs, TIMP-1, TIMP-2 and TIMP-3 messengers were detected in all conditions. Our analysis was also extended to the molecules which expression may be responsible primarily for changes in the proMMP-2 extracellular pool. In fact, MMP-14 (Membrane type 1 MMP), the major activator of the enzyme, was present as mRNA. On the other hand, we investigated the expression of thrombospondins 1 and 2, which are matricellular proteins involved in the process of clearance of MMP-2 from the extracellular space [29]. As shown in figure 7, both molecules were expressed by HFL-1 cells.

### Semi-Quantitative evaluation of expression of MMP-2 mRNA

**Figure 8:**
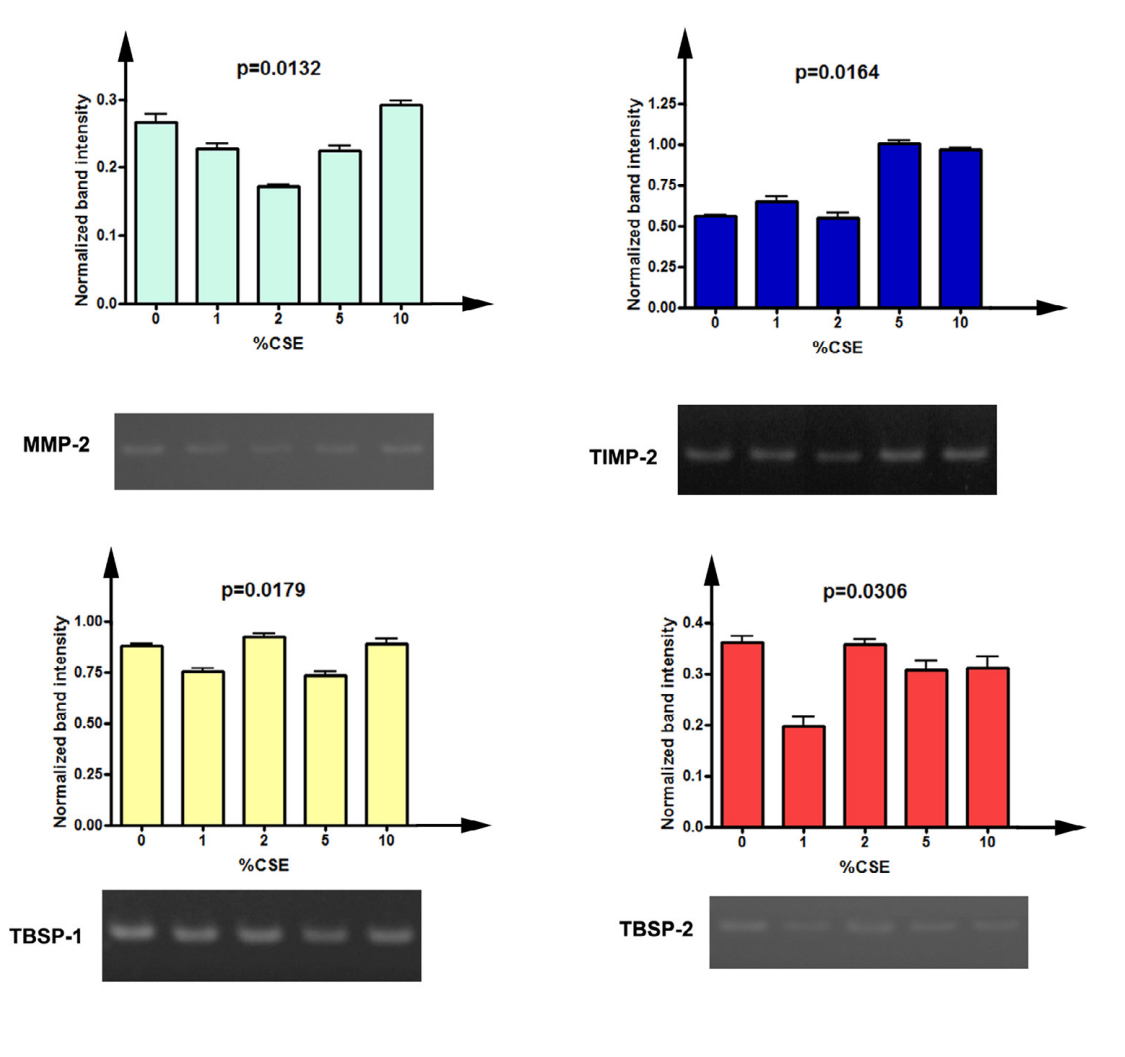
**Semi-quantitative densitometric analysis of the expression of MMP-2, TIMP-2 and thrombospondins after CSE exposure**. Data are represented as mean, with the indication of standard deviation, of three replicate experiments. Values were normalized for beta actin expression. The significance of differences has been evaluated with the Kruskal-Wallis test. A) Expression levels of MMP-2 (cycles = 30). B) Expression levels of TIMP-2 (cycles = 30). C) Expression levels of thrombospondin-1 (cycles = 30). D) Expression levels of thrombospondin-2 (cycles = 32).

In order to determine the regulatory effects of CSE on transcriptional levels of MMP-2, as well as its specific inhibitor TIMP-2 and the two thrombospondin molecules, we performed a semi-quantitative evaluation of the mRNAs levels by densitometry, with comparison to the housekeeping gene beta actin [16]. The results of this analysis are depicted in figure 8. As visible in figure 8A, levels of MMP-2 mRNA were significantly lowered after CSE exposures (p = 0.0132), even if at 10% CSE the messenger level was similar to that of untreated cells. Moreover, Dunn's post test indicated that for 2% CSE the difference with respect to untreated cells was statistically significant (p > 0.05). Interestingly, TIMP-2 expression levels were markedly elevated at both 5% and 10% CSE (p = 0.0164), thus suggesting that CSE should inversely regulate the transcriptional levels of both enzyme and inhibitor. Thrombospondin-1 expression varied slightly but significantly (p = 0.0179). Finally, Thrombospondin-2 levels were significantly lower after CSE exposure (p = 0.0306).

### SM-PCR analysis of MMP-2 expression following CSE exposure

**Figure 9:**
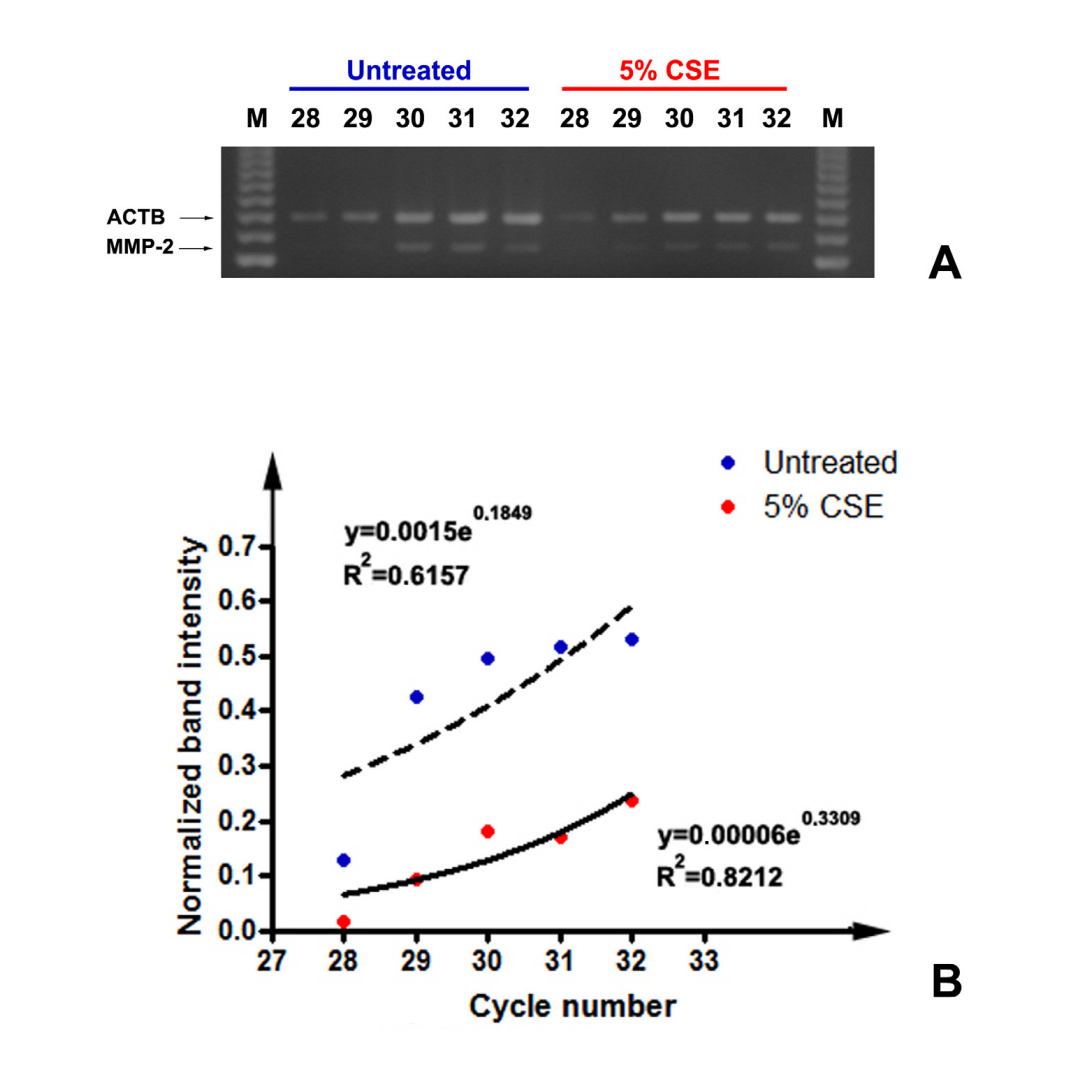
**SM-PCR analysis of MMP_2 expression after 5% CSE exposure**. Representative plots of normalized data vs. cycle numbers fit with an exponential curve for untreated and 5% CSE-exposed cells.

In order to confirm the variations in MMP-2 expression observed with semi-quantitative densitometry after RT-PCR, we applied the technique of semi-quantitative multiplex PCR (SM-PCR) [25-27]. As visible in figure 9, we co-amplified the PCR products corresponding to MMP-2 and beta actin, over a range of cycles starting from 28 (the minimum required for the detection of MMP-2, as determined in control experiments). Primers for beta actin were added after two cycles, in order to keep the reference PCR product in the exponential phase of PCR. As shown in figure 9B, the densitometry data obtained after normalization of band intensity for the reference gene, were plotted to extrapolate the fitted exponential curve. R^2 ^coefficients were calculated for both curves and confirmed the fitting of the curves with the exponential one. As visible and confirming the previously described result, MMP-2 mRNA expression was lower after 5% CSE exposure, over the exponential phase of the PCR reaction.

## Discussion

Targeting of proteolytic activities is considered one of the promising therapeutic strategies for lung inflammatory diseases, as COPD and asthma [30,31]. Since multiple cell types (both resident and migrating ones) are involved in the genesis and maintenance of an inflamed state, several may be also the possible targets of a therapeutic approach aiming to the restoration of tissue homeostasis by the normalisation of the proteolysis/antiproteolysis balance. Multiple reports linked an imbalance of extracellular lytic activity to the progression of inflammatory lung diseases [32,33]. Fibroblasts are the main cell type of lung parenchyma: these cells secrete ECM molecules as well as proteinases, and physiologically regulate matrix homeostasis. Moreover, when subject to a damaging stimulus, as cigarette smoke, these cells are also capable to release chemotactic factors (in order to recruit monocytes and neutrophils) together with proinflammatory mediators, thus creating a proinflammatory microenvironment [15,17]. Present data strongly suggest that, in HFL-1 lung fibroblasts CSE exerts a significant negative modulator effect on pro-MMP-2 extracellular activity. The decrease in activity levels of the enzyme in the extracellular space should be due to the reduced transcription, as strongly suggested by semi-quantitative analysis of expression, with significant reduction at 2% CSE, as shown by Dunn's post test. Moreover, at doses of 5% and 10% CSE was observed an increase of TIMP-2 mRNA. Therefore, present results strongly suggest that cigarette smoke extract may exert a direct effect on the proteinase/antiproteinase balance in human lung fibroblasts, since it caused a significant decrease of the enzyme activity and expression, as well as an increase in the expression of its specific inhibitor. On the other hand, CSE appeared to cause minor effects on both thrombospondins expression with a global trend of decrease which reached statistical significance. Hence, CSE exposure of human lung fibroblasts ultimately leads to a downregulation of MMP-2 expression and an upregulation of TIMP-2. To our knowledge, this is the first study linking CSE exposure of human lung fibroblasts to a significant reduction in extracellular MMP-2 activity. Fibroblasts constitutively secrete MMP-2, indeed in physiological conditions these cells produce both ECM molecules and ECM-degrading enzymes. The negative regulation of MMP-2 extracellular activity in CSE exposed cells is a result which may integrate the current models of COPD progression, which are focused on an increase of some extracellular lytic activities (as shown for MMP-12 and MMP-9 produced by inflammatory cells) [14,34]. Ex vivo studies recently showed a link between tobacco smoking and lytic activity of gelatinases [32,35]. Interestingly, in a recent study, Wong et al [36] have shown that exposure to cigarette smoke exerted different effects on fibroblasts, such as delay of wound repair, decrease in cell migration and increase in cell survival, potentially leading to excessive deposition of matrix and tissue fibrosis. Therefore, results of present study are in good agreement with this model, since lower MMP-2 activity levels in the pericellular space may be linked to both decrease in cell motility and excessive ECM deposition. Moreover, cytotoxicity tests strongly suggested that low doses of CSE should result in an increased proliferation of cells. More interestingly, MMPs, and in particular gelatinase A, may also have a limiting role in inflammatory processes [37,38]. In fact, some chemokines may be inactivated by MMPs, thereby restricting monocyte migration towards sites of injury. Therefore, in a normal inflammatory process some MMPs (as macrophage-derived MMP-9) may initially promote cell influx to damaged areas, while at the end other enzymatic activities belonging to the same family (and displaying a characteristic substrate overlap) may be required to degrade proinflammatory mediators to stop the process. When inflammation persists, as in COPD, the cellular interplay is affected, and our data, showing a reduction in fibroblast-secreted MMP-2 activity, may suggest an additional type of contribution of lung structural cells to the phenomenon of persistence of tissue inflammation. Smoke exposure of lung fibroblasts may result in a decrease of bioavailable MMP-2, both in the free proenzymatic form, and, as reported in this work for the first time, for its higher molecular weight complexes. Loss of gelatinase A activity may then lead to an impaired tissue repair. Since remodelling of the lung ECM is a process which involves several players at the cellular level and a consistent number of effectors molecules (of which MMPs constitute a key group), in our opinion further studies should be needed to better detail, in lung fibroblasts, but also in epithelial cells, the effects of CSE on MMP/TIMP balance and, conversely, on maintenance of ECM features. This point should become of great interest, since inhibition of proteolytic enzymes is being considered as a promising therapeutic strategy for COPD treatment. Therefore, it should be important to determine which proteolytic enzymes must be inhibited and which may be otherwise important to accomplish a full tissue repair after the inflammatory process has taken place.

## Conclusion

The present work investigates for the first time the effects of smoke exposure on activity levels of MMP-2 in human lung fibroblasts. Interestingly, cells react to this stimulus, at doses which do not cause any significant increase in cell mortality, with a reduction of extracellular levels of MMP-2. We showed with different techniques that this effect is not due neither to any direct inhibition of gelatinolytic activity by CSE, or to aspecific cytotoxic effects of smoke extract. Interestingly, CSE exposure caused a decrease in MMP-2 mRNA, together with a significant increase of TIMP-2 expression. Therefore, this work integrates the present view of matrix remodelling in COPD, where besides to the increased activity levels of macrophage-derived MMP-9 and MMP-12, fibroblasts may contribute with a decrease of MMP-2 activity. Since MMP inhibition is being considered as a promising therapeutic strategy for lung chronic inflammatory diseases, it should be important to determine which MMPs must be inhibited and which may be otherwise important to accomplish a full tissue repair after the inflammatory process has taken place.

## Abbreviations

CSE, cigarette smoke extract

COPD, chronic obstructive pulmonary disease

ECM, extracellular matrix

GAPDH, glyceraldehyde-3-phosphate dehydrogenase

HFL, human foetal lung

MMP, matrix metalloproteinase

PMSF, Phenylmethylsulphonylfluoride

RT-PCR, reverse transcription-polymerase chain reaction

SM-PCR, Semi-quantitative multiplex polymerase chain reaction

TIMP, tissue inhibitor of metalloproteinases

TBSP, thrombospondin

YWHAZ, tyrosine 3-monooxygenase/tryptophan 5-monooxygenase activation protein, zeta polypeptide.

## Competing interests

The author(s) declare that they have no competing interests.

## Authors' contributions

GLR assisted in the study design, carried out the zymographic and densitometric analyses and drafted the manuscript. RA carried out cell cultures, viability assays, RNA extraction and RT-PCR analyses. FM carried out western blotting and chromatographic analyses. FF assisted in study design, statistical analyses and interpretation of data. FC carried out statistical analyses and inhibition assays. GZ conceived the study, supervised study design and assisted manuscript drafting. All authors read and approved the final manuscript.
